# A nutritional supplement containing zinc during preconception and pregnancy increases human milk zinc concentrations

**DOI:** 10.3389/fnut.2022.1034828

**Published:** 2023-01-10

**Authors:** Soo Min Han, Surabhi Devaraj, José G. B. Derraik, Mark H. Vickers, Fang Huang, Stephane Dubascoux, Keith M. Godfrey, Shiao-Yng Chan, Wei Wei Pang, Sagar K. Thakkar, Wayne S. Cutfield, NiPPeR Study Group, Benjamin B Albert

**Affiliations:** ^1^Liggins Institute, The University of Auckland, Auckland, New Zealand; ^2^Nestlé Research, Société des Produits Nestlé SA, Singapore, Singapore; ^3^Department of Women's and Children's Health, Uppsala University, Uppsala, Sweden; ^4^Environmental-Occupational Health Sciences and Non-Communicable Diseases Research Group, Research Institute for Health Sciences, Chiang Mai University, Chiang Mai, Thailand; ^5^Department of Paediatrics: Child and Youth Health, Faculty of Medical and Health Sciences, School of Medicine, University of Auckland, Auckland, New Zealand; ^6^Nestlé Research, Société des Produits Nestlé SA, Beijing, China; ^7^Nestlé Research, Société des Produits Nestlé SA, Lausanne, Switzerland; ^8^MRC Lifecourse Epidemiology Unit, University of Southampton, Southampton, United Kingdom; ^9^NIHR Southampton Biomedical Research Centre, University Hospital Southampton, NHS Foundation Trust, Southampton, United Kingdom; ^10^Department of Obstetrics and Gynaecology, Yong Loo Lin School of Medicine, National University of Singapore and National University Health System, Singapore, Singapore; ^11^Singapore Institute for Clinical Sciences, Agency for Science, Technology and Research, Singapore, Singapore; ^12^A Better Start – National Science Challenge, The University of Auckland, Auckland, New Zealand

**Keywords:** human milk, minerals, pregnancy, supplement, zinc

## Abstract

**Introduction:**

During pregnancy and lactation minerals such as zinc are required to support maternal and infant health. Zinc is involved in various cellular processes, with requirements increasing in pregnancy and lactation. In the setting of a randomized trial, we investigated the effects on human milk (HM) zinc concentrations of a micronutrient-containing supplement including zinc in the intervention (but not control) group, started preconception and taken throughout pregnancy until birth. Additionally, we characterized longitudinal changes in HM concentrations of zinc and other minerals (calcium, copper, iodine, iron, magnesium, manganese, phosphorus, potassium, selenium, and sodium).

**Methods:**

HM samples were collected across 7 time points from 1 week to 12 months from lactating mothers from Singapore (*n* = 158) and New Zealand (*n* = 180). HM minerals were quantified using sector field inductively coupled plasma mass spectrometry. Potential intervention effects on HM mineral concentrations were assessed using linear mixed models with a repeated measures design and time-weighted area-under-the-curve analyses.

**Results:**

Over the first 3 months of lactation, HM zinc concentrations were 11% higher in the intervention group compared to the control group (*p* = 0.021). Higher HM zinc concentrations were most evident at 6 weeks of lactation. The intervention had no effect on HM concentrations of other minerals, which were not differently supplemented to the control and intervention groups. Temporal changes in HM minerals over 12 months of lactation were studied in the New Zealand mothers; HM zinc and copper concentrations progressively decreased throughout 12 months, while iron, potassium, sodium, and phosphorus decreased until 6 months then plateaued. HM calcium and magnesium initially increased in early lactation and iodine remained relatively constant throughout 12 months. HM manganese and selenium fell over the initial months of lactation, with a nadir at 6 months, and increased thereafter. The contrasting patterns of changes in HM mineral concentrations during lactation may reflect different absorption needs and roles at different stages of infancy.

**Discussion:**

Overall, this study indicates that HM zinc concentrations are influenced by maternal supplementation during preconception and pregnancy. Further studies are required to understand the associations between HM zinc and other minerals and both short- and long-term offspring outcomes.

**Trial registration:**

ClinicalTrials.gov, identifier: NCT02509988, Universal Trial Number U1111-1171-8056. Registered on 16 July 2015. This is an academic-led study by the EpiGen Global Research Consortium.

## 1. Introduction

Adequate mineral status during pregnancy and lactation is essential for the health of the mother and optimal growth and development of the infant (1). Minerals play important roles in brain development (iron, zinc, copper, and iodine), bone health (calcium, phosphorus, and magnesium), and thyroid hormone metabolism (selenium, zinc, and iodine) (1, 2).

Mineral requirements increase during pregnancy to support the physiological changes in the mother and the growing fetus. For example, an ~18–36% additional zinc is required during pregnancy (3), the daily recommended intake being 7–15 mg (4–6). Zinc deficiency in pregnancy is prevalent worldwide, especially in countries with poor nutrition (7, 8). In New Zealand, 18.8% of women aged 19–30 years were estimated to have inadequate zinc intake (9). In Singapore, 19% of pregnant women (at 26–28 weeks' gestation) were estimated to be zinc deficient (10). This has been associated with adverse complications such as preterm birth, intrauterine growth restriction, and low infant birth weight (7, 11). In women with a risk of zinc deficiency, supplementation may be beneficial (12–14).

Human milk (HM) is the major source of zinc for newborn infants, who require 2–4 mg/day (aged 0–6 months) and 3–5 mg/day (aged 7–12 months) (4, 6). As such, physiological mechanisms to maintain adequate zinc status of the mother and the infant, *via* HM, continues during lactation. This is associated with mobilization of maternal zinc pools from involuting tissues as well as the trabecular bone (3, 14). In HM, zinc has been identified to be bound to protein ligands, casein and serum albumin associated with 14% and 28% of total HM zinc, respectively (15). Zinc is involved in various cellular processes, enzyme functions, and immune functions (16). Zinc has been associated with growth (17, 18), the immune system (19, 20), and cognitive development (21) in infants. Therefore, ensuring optimal maternal zinc status during lactation is crucial for supporting infant growth.

The Nutritional Intervention Preconception and During Pregnancy to Maintain Healthy Glucose Metabolism and Offspring Health (NiPPeR) study was designed to investigate the effects of an enhanced micronutrient supplement during preconception and pregnancy on maternal pregnancy outcomes and infant growth (22). The control and intervention supplements both contained micronutrients (i.e., calcium, iron, and iodine) that are part of common pregnancy supplements. For example, calcium plays a role in fetal bone formation, and enzyme and hormone functioning, and supplementation during pregnancy has been associated with reduced risks of preeclampsia and preterm birth (23). Iodine is essential for maternal and fetal thyroid hormone production, and its deficiency increases the risks of infant mortality and intellectual impairment (24, 25). Iron deficiency during pregnancy may lead to anemia in mothers, which has been associated with low birth weight and preterm birth (26). The intervention supplement contained additional micronutrients, including zinc. Overall, understanding the factors that influence HM mineral concentrations, such as maternal micronutrient supplement use and lactation stage, is essential to ensure optimal nutrition status in breastfeeding mothers and breastfed infants.

The aim of the present study was to assess the effects of the intervention supplement taken during preconception and pregnancy on subsequent HM zinc and other mineral concentrations. Moreover, the longitudinal changes in HM minerals (zinc, calcium, copper, iodine, iron, magnesium, manganese, phosphorus, potassium, selenium, and sodium) in the first year of lactation were analyzed. We hypothesized that (i) zinc supplementation during preconception/pregnancy would influence HM zinc concentrations during lactation, and (ii) HM concentrations of zinc and other minerals would have differing patterns of change over 12 months of lactation.

## 2. Materials and methods

### 2.1. Study design

The detailed protocol for the NiPPeR study (ClinicalTrials.gov, identifier: NCT02509988, Universal Trial Number U1111-1171-8056; registered on 16 July 2015) has been published previously (22). In brief, the NiPPeR study was a double-blind, randomized controlled trial investigating the effects of a nutritional supplement taken from preconception and during pregnancy on maternal pregnancy and infant outcomes. The control supplement comprised of standard amounts of micronutrients that are present in supplements commonly used during pregnancy including calcium, iron, iodine, folic acid, and β-carotene (Table 1). In addition to these nutrients, the NiPPeR intervention supplement contained vitamins B_2_, B_6_, B_12_, and D, as well as zinc, myo-inositol, and probiotics (Table 1). Importantly, zinc was the only mineral present in the intervention supplement, but not in the control supplement. The study supplements were packaged as a powder in sachets, and were taken twice daily as a drink reconstituted with water. Adherence to the study drinks was ascertained by sachet counting, with good adherence defined as at least 60% of the sachets taken (27). The study was conducted in Southampton (UK), Singapore, and Auckland (New Zealand), with ethics approval obtained at each site [Southampton—Health Research Authority National Research Ethics Service Committee South Central Research Ethics Committee (15/SC/0142); Singapore—the National Healthcare Group Domain Specific Review Board (2015/00205); and New Zealand—Northern A Health and Disability Ethics Committee (15/NTA/21)]. All participants provided written informed consent.

**Table 1:** Detailed nutrient composition of the intervention and control drinks in the NiPPeR study.

**Group**	**Nutrient**	**Intervention**	**Control**	**Daily dose**	**Recommended range^#^**
Minerals	Calcium (as calcium-L-lactate)	✓	✓	150 mg	700–1,300 mg
	Iodine (as potassium iodide)	✓	✓	150 μg	140–220 μg
	Iron (as ferric pyrophosphate)	✓	✓	12 mg	14.8–27 mg
	Zinc (as zinc glycinate chelate)	✓	***x***	10 mg	7–15 mg
Vitamins	A (β-carotene)	✓	✓	720 μg	700–750 μg
	B_2_ (riboflavin)	✓	***x***	1.8 mg	1.38–1.46 mg
	B_6_ (pyridoxine)	✓	***x***	2.6 mg	1.2–1.9 mg
	B_9_ (folic acid)	✓	✓	400 μg	300–600 μg
	B_12_ (cobalamin)	✓	***x***	5.2 μg	1.5–2.6 μg
	D_3_ (cholecalciferol)	✓	***x***	400 IU (10 μg)	5–10 μg
Other	Myo-inositol	✓	***x***	4 g	n/a
	*Lactobacillus rhamnosus* ^*^	✓	***x***	>1 × 10^9^ CFU	n/a
	*Bifidobacterium animalis* ssp. *lactis*^†^	✓	***x***	>1 × 10^9^ CFU	n/a

# Recommended ranges for daily intake during pregnancy according to the reference nutrient intake for the UK (4), recommended dietary allowance for Singapore (5), and recommended daily intake for New Zealand (6).

* NCC 4007 (CGMCC 1.3724).

† NCC 2818 (CNCM I-3446).

CFU, colony-forming units; n/a, not applicable.

### 2.2. Study participants

Participants were recruited by self-referral after study information was disseminated through local and social media advertisements. The full inclusion, exclusion, and withdrawal criteria have been reported previously (22), and are provided in Supplementary Table 1. Briefly, women aged 18–38 years who were planning to conceive within 6 months were eligible for the study and withdrawn if they had not conceived within 12 months. Eligible participants were randomized in a 1:1 ratio to either the control or the intervention group through the electronic study database (22), and stratified by site and ethnicity to ensure balanced allocation of participants.

### 2.3. Human milk sample collection

**Figure 1:**
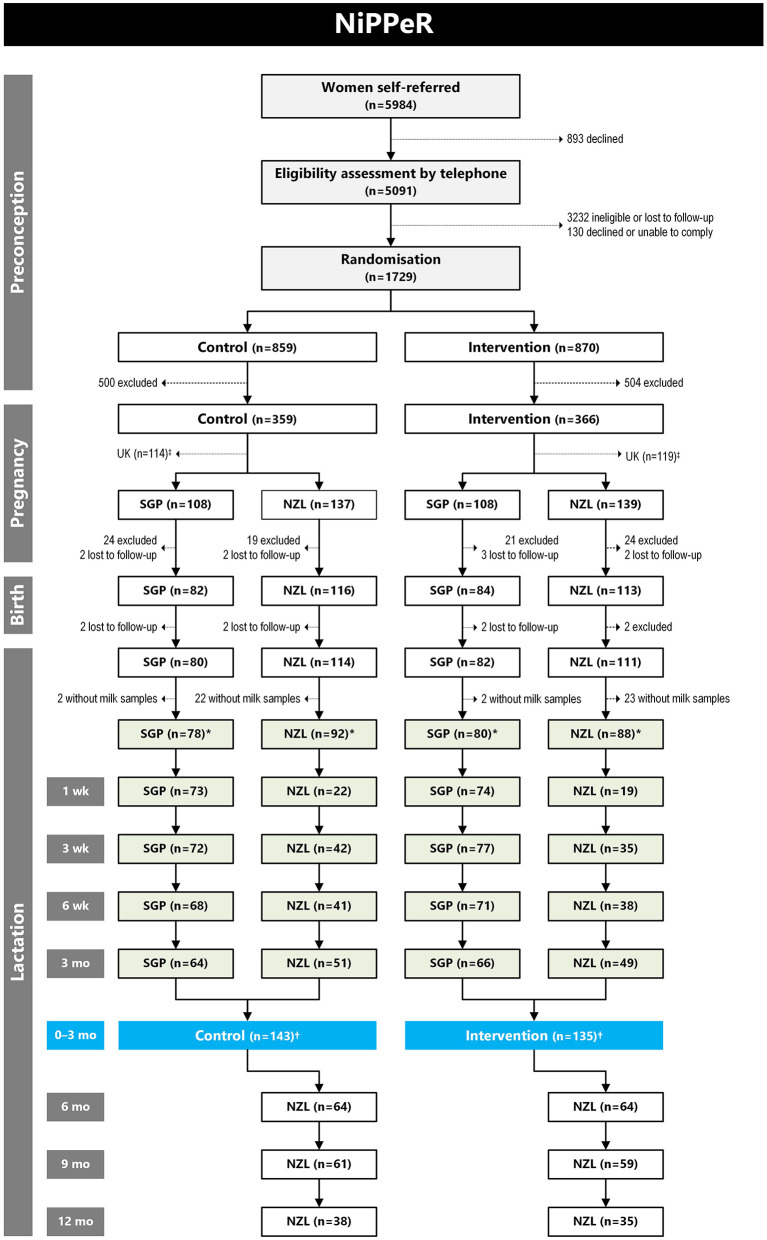
CONSORT diagram for number of human milk (HM) samples analyzed for mineral concentrations in the NiPPeR study. Reasons for exclusion during the preconception phase have been published previously (27), while reasons for exclusion during pregnancy and birth in Singapore (SGP) and New Zealand (NZL) are provided in Supplementary Table 2. There were no HM samples collected in the United Kingdom (UK), so all participants from that site were excluded from this diagram. *Number of participants who provided at least one HM sample during 12 months of lactation. ^†^Number of participants who provided at least one HM sample during the first 3 months of lactation.

HM samples were collected only in Singapore for 3 months of lactation (from July 2016 to March 2019) and New Zealand for up to 12 months of lactation (from May 2017 to November 2019) (Figure 1). Samples were collected at 1 week ± 3 days, 3 weeks ± 5 days, 6 weeks ± 5 days, and 3 months ± 10 days (4 time points); in New Zealand, there were additional HM collections at 6 months ± 14 days, 9 months ± 14 days, and 12 months ± 14 days (7 time points overall). In Singapore, samples could only be collected until 3 months due to logistical constraints. HM samples were collected in the morning, and mothers were asked to refrain from breastfeeding for 2 h prior to collection from the unilateral breast from where samples would be collected. Whole HM samples were collected from a single breast using an Ameda Lactaline breast pump (Ameda, Inc, Murarrie, Australia). The breast was pumped for 15 min or until fully emptied, under the supervision of a trained staff. Soon after collection, HM samples were vortexed for homogenization, divided into aliquots and then stored at −80°C until analysis. HM samples were not collected if the mother refused, had ceased breastfeeding, her milk supply was low, or there were complications with milk expression. The total number of samples collected at each time point are outlined in Figure 1. The number of participants with longitudinal samples to 3 months of lactation is summarized in Table 2, and to 12 months of lactation in New Zealand in Table 3.

**Table 2:** Number of longitudinal human milk (HM) samples collected per participant in the first 3 months of lactation in Singapore and New Zealand.

**HM samples (*n*)**	**Control**			**Intervention**		
	**Overall**	**Singapore**	**New Zealand**	**Overall**	**Singapore**	**New Zealand**
0	27	–	27	33	–	33
1	22	3	19	16	2	14
2	25	9	16	18	7	11
3	23	8	15	27	12	15
4	73	58	15	74	59	15
Total	170	78	92	168	80	88

**Table 3:** Number of longitudinal human milk (HM) samples collected in New Zealand during 12 months of lactation.

**HM samples (*n*)**	**Control**	**Intervention**
1	21	16
2	17	17
3	10	20
4	13	7
5	11	12
6	13	11
7	7	5
Total	92	88

### 2.4. Human milk mineral quantification

HM mineral quantification was carried out by ALS Scandinavia AB (Luleå, Sweden). HM calcium, cobalt, copper, iron, potassium, magnesium, manganese, sodium, nickel, phosphorus, selenium, and zinc were quantified using sector field inductively coupled plasma mass spectrometry (SF-ICP-MS), ELEMENT 2 (Thermo, Bremen, Germany) equipped with an ASX 500 sample changer (CETAC Technologies Inc., Omaha, USA), using a modified version of methods described by Rodushkin et al. (28, 29), with the sample intake for the microwave-assisted acidic decomposition reduced to 0.2 mL. Briefly, HM samples (0.2 mL), were transferred in perfluoroalkoxy polymer lined vessels and mineralized with a microwave oven (MDS-2000, CEM Corporation, Matthews, USA) using analytical grade nitric acid (Merck, Darmstadt, Germany) after additional purification by sub-boiling distillation in a quartz still. After mineralization, the resulting solutions were diluted with Milli-Q water (Millipore Milli-Q, Bedford, USA) and spiked with internal standard solution containing scandium, indium, and lutetium. Iodine was also quantified using SF-ICP-MS ELEMENT 2 with an ASX 500 sample changer, but following the instrumental method by Engström et al. (30). Sample preparation was slightly modified from Krachler et al. (31) using the alkaline reagent composition given by Engström et al. (30) and a dilution factor of 1:50. Briefly, prior to ICP-MS analysis, HM samples were diluted (with a dilution factor of 1:50) with alkaline diluent containing 0.01 M ammonia (Suprapur, Merck), 0.2 mM (NH_4_)_2_EDTA (Fluka) and 0.07% Triton X-100 (Merck). All intra- and inter-assay coefficients of variation were < 10%.

### 2.5. Statistical analyses

Mineral concentration measurements below the lower level of quantification (LLoQ) were assigned a value of 0.5 × LLoQ (Supplementary Table 3). To minimize the removal of values from the data set, we adopted a conservative approach defining extreme values (i.e., outliers) as measurements outside the mean ± 5 standard deviations (SD) range. There were no values below mean – 5 SD range, but for some minerals there were a few values greater than the mean + 5 SD classified as extreme values (i.e., >99.99997th percentile) (Supplementary Table 3) and removed from analyses. It was not possible to undertake reliable statistical analyses on cobalt and nickel as a large proportion of values were below the LLoQ (41.4 and 83.0%, respectively). For all other minerals, data were log-transformed to approximate a normal distribution, then back-transformed for reporting.

Potential intervention effects on HM mineral concentrations were only examined on the samples collected in the first 3 months of lactation, which were collected in both Singapore and New Zealand. In a sensitivity analysis this was also assessed in a subgroup of participants who provided consecutive samples across the 4 time points in the first 3 months. Data were analyzed using linear mixed models with a repeated measures design. Parameters included were randomization group, visit, their interaction term (group^*^visit), and study site, as well as adherence to the study protocol, maternal pre-pregnancy body mass index (BMI), and gestational age at birth as covariates. The participant's study ID was also included as a random factor to account for the multiple measurements on the same individual (non-independence). If the interaction term was statistically significant, between-group comparisons were only reported on a per-visit basis.

The time-weighted area-under-the-curve (TwAUC) was also calculated for each participant who had at least 3 valid HM measurements within the first 3 months of lactation, using the following formula:


(1) $$\begin{array}{c} TwAUC=\frac{AUC}{age_{t}-age_{0}} \end{array}$$

where *age*_0_ and *age*_*t*_ were the infant's ages when the first and last measurements used in the AUC were collected, respectively. TwAUC data were analyzed using general linear models adjusted for study site, adherence, maternal pre-pregnancy BMI, and gestational age at birth.

Subgroup analyses were also performed to examine potential treatment effects over the first 3 months of lactation separately for Singapore and New Zealand. Temporal changes in HM minerals from 1 week to 12 months of lactation were plotted and reported for the New Zealand site only. These were also examined in a subgroup of New Zealand participants who provided HM samples for at least five out of six time points between 3 weeks and 12 months.

Study outcomes are reported as the back-transformed least-squares means (i.e., adjusted means) for each group or the adjusted mean differences (aMD) between groups, and their respective 95% confidence intervals (CI). Note that the aMD for back-transformed values represent proportional differences between groups. Statistical analyses were carried using SAS version 9.4 (SAS Institute Inc., Cary, NC, USA) and graphs created with GraphPad Prism version 8.2.1 (GraphPad Software, San Diego, California USA). All statistical tests were two-sided with significance maintained at *p* < 0.05, without adjustments for multiple comparisons or imputation of missing values.

## 3. Results

### 3.1. Study population

At Singapore and New Zealand sites combined, 387 participants continued to postpartum stage of the study, of which 338 participants (87.3%) provided at least one HM sample during the study period (Figure 1). Maternal demographic and pre-pregnancy BMI characteristics were similar in control and intervention groups (Table 4), noting that participants were mostly Chinese in Singapore and Caucasian in New Zealand (Supplementary Table 4). Adherence to the study drinks was high and averaged at about 87% consumption for both groups. The mean (±SD) duration of supplementation was 405 ± 105 days in the control group and 393 ± 98 days in the intervention group. Passive smoking during pregnancy was more common among controls than in the Intervention group (19.4 vs. 9.5%, respectively; *p* = 0.013) [comparison made with a Fisher's exact test]. Other pregnancy and birth outcomes were also similar between the two groups overall (Table 4) and within sites (Supplementary Table 4). The characteristics for the subgroup who provided HM samples were similar to the total group of participants from Singapore and New Zealand sites who continued to the postpartum stage of the study (Supplementary Table 5).

**Table 4:** Baseline and perinatal characteristics of participants in the NiPPeR study who provided at least one human milk sample in 12 months of lactation.

	**Overall (*****n*** = **338)**	
	**Control**	**Intervention**
*n*	170 (50.3%)	168 (49.7%)
Adherence (%)	87.4 ± 11.2	86.9 ± 13.4
Duration of supplementation (days)	405 ± 105	393 ± 98
Age at delivery (years)	31.9 ± 2.9	32.4 ± 3.2
Maternal pre-pregnancy BMI (kg/m^2^)	24.4 ± 5.2	23.4 ± 4.4
**Ethnicity**		
Caucasian	70 (41.2%)	67 (39.9%)
Chinese	70 (41.2%)	69 (41.1%)
South Asian	10 (5.9%)	10 (6.0%)
Malay	10 (5.9%)	10 (6.0%)
Other	10 (5.9%)	12 (7.1%)
**Maternal pre-pregnancy BMI status**		
Underweight or normal weight	100 (58.8%)	103 (61.3%)
Overweight	41 (24.1%)	48 (28.6%)
Obesity	29 (17.1%)	16 (9.5%)
Missing	–	1 (0.6%)
**Highest level of education**		
Bachelor's degree or higher	137 (80.6%)	136 (81.0%)
Lesser qualification^*^	33 (19.4%)	32 (19.0%)
**Household income quintile**		
5 (lowest)	4 (2.4%)	1 (0.6%)
4	12 (7.1%)	16 (9.5%)
3	44 (25.9%)	43 (25.6%)
2	60 (35.3%)	55 (32.7%)
1 (highest)	44 (25.9%)	43 (25.6%)
Missing	6 (3.5%)	10 (6.0%)
**Smoking during pregnancy**		
None	134 (78.8%)	148 (88.6%)
Passive	33 (19.4%)	16 (9.6%)
Active	3 (1.8%)	3 (1.8%)
Missing	–	1 (0.6%)
**GDM**		
No GDM	126 (74.1%)	125 (74.4%)
GDM	42 (24.7%)	43 (25.6%)
Missing	2 (1.2%)	–
No	167 (98.8%)	165 (98.2%)
Yes	2 (1.1%)	3 (1.8%)
Missing	1 (0.6%)	–
**Mode of delivery**		
Vaginal delivery	125 (73.5%)	119 (70.8%)
Cesarean section	44 (25.9%)	49 (29.2%)
Missing	1 (0.6%)	–
**Infant gestational age**		
Gestational age (weeks)	39.1 ± 1.6	39.2 ± 1.5
Preterm	14 (8.2%)	11 (6.5%)
Term or post-term	156 (91.8%)	157 (93.5%)
**Infant birth weight**		
Birth weight (kg)	3.24 ± 0.54	3.23 ± 0.53
Appropriate for gestational age	144 (84.7%)	143 (85.1%)
Small for gestational age	16 (9.4%)	19 (11.3%)
Large for gestational age	10 (5.9%)	6 (3.6%)
**Parity**		
Primiparous	114 (67.1%)	95 (56.5%)
Multiparous	56 (32.9%)	73 (43.5%)
**Infant sex**		
Male	76 (44.7%)	79 (47.0%)
Female	94 (55.3%)	89 (53.0%)

Data are n (%) or mean ± standard deviation (SD). Adherence to the study protocol was determined by sachet counting. Duration of supplementation calculated by counting the number of days from randomization date to delivery date. Body mass index (BMI) status was defined using ethnic-specific thresholds for BMI categories: for Asians, under or normal weight < 23.0 kg/m^2^, overweight 23.0–27.49 kg/m^2^, obesity ≥27.5 kg/m^2^; for non-Asians, under or normal weight < 25.0 kg/m^2^, overweight 25.0–29.99 kg/m^2^, obesity ≥30.0 kg/m^2^. Gestational diabetes (GDM) was defined by International Association of Diabetes and Pregnancy Study Groups criteria (59). Gestational age was determined using a pre-specified algorithm as previously described (60) with preterm defined as birth < 37 weeks of gestation, and term or post-term as birth at ≥37 weeks of gestation. ^*^Including incomplete and complete high school qualifications, and other tertiary level qualifications below bachelors (e.g., diploma or certificate).

### 3.2. Impact of intervention on zinc and other minerals

The mean HM zinc concentrations over the first 3 months of lactation were 11% higher in the intervention than in the control group (*p* = 0.021; Table 5), with a similar difference observed for the TwAUC (*p* = 0.022;

**Table 5:** Mineral concentrations in human milk (HM) over the first 3 months of lactation in the intervention and control groups.

**Mineral**	**Intervention**	**Control**	**aMD**	***p*-value**
Zn (μg/L)^*^	2,490 (2,338, 2,652)	2,246 (2,111, 2,388)	1.109 (1.016, 1.210)	**0.021**
Calcium (mg/L)^†^	286 (277, 296)	287 (278, 297)	0.996 (0.951, 1.043)	0.868
Copper (μg/L)	386 (373, 400)	387 (374, 401)	0.997 (0.949, 1.048)	0.910
Iodine (μg/L)^†^	113 (106, 122)	117 (109, 125)	0.972 (0.883, 1.070)	0.562
Iron (mg/L)^†^	0.25 (0.24, 0.27)	0.25 (0.23, 0.26)	1.028 (0.952, 1.110)	0.485
Magnesium (mg/L)	29.1 (28.3, 29.9)	28.6 (27.8, 29.3)	1.018 (0.981, 1.057)	0.339
Manganese (μg/L)	2.55 (2.39, 2.72)	2.42 (2.27, 2.58)	1.053 (0.963, 1.152)	0.257
Phosphorus (mg/L)	153 (148, 158)	150 (145, 155)	1.021 (0.975, 1.068)	0.375
Potassium (mg/L)	544 (534, 555)	548 (539, 558)	0.993 (0.968, 1.019)	0.584
Selenium (μg/L)	16.5 (16.0, 17.0)	16.6 (16.2, 17.1)	0.990 (0.952, 1.030)	0.626
Sodium (mg/L)	143 (135, 152)	148 (140, 157)	0.964 (0.888, 1.047)	0.388

^*^Mineral present only in the intervention drink. ^†^Minerals present in both control and intervention drinks. Data are the least-squares mean (i.e., adjusted mean) for each group or the adjusted mean difference (aMD) and respective 95% confidence intervals derived from repeated measures analyses, adjusted for visit, an interaction term (group^*^visit), study site, adherence, maternal pre-pregnancy body mass index, and gestational age at birth. All data have been log-transformed to approximate a normal distribution, and then back-transformed, so the aMD represents a proportional difference between groups (i.e., intervention vs. control). Bold font indicates a statistically significant difference between groups (at p < 0.05).

**Figure 2:**
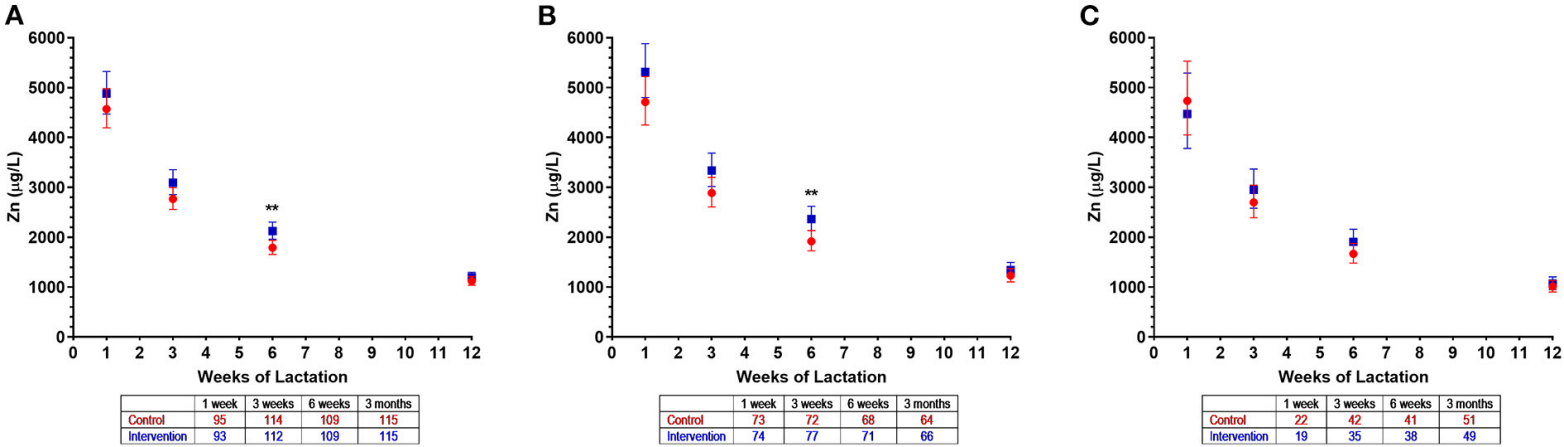
Zinc concentrations in human milk of control (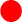) and intervention (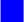) groups in the NiPPeR study during the first 3 months of lactation: **(A)** Overall, **(B)** Singapore, and **(C)** New Zealand. Data are the least-squares means (i.e., adjusted means) for each group, adjusted for visit, an interaction term (group*visit), study site, adherence, maternal pre-pregnancy body mass index, and gestational age at birth; error bars represent the respective 95% confidence intervals. *p < 0.01 for the difference between intervention and control groups at a given time point. The number of HM samples per group analyzed at a given time point are provided in the tables below the *x* axes.

Table 6). When zinc concentrations at individual visits were examined, the difference between groups was most evident at 6 weeks: 2,126 μg/L (95% CI 1,962, 2,304) and 1,790 μg/L (95% CI 1,654, 1,938) in the intervention and control groups, respectively (*p* = 0.003; Figure 2A). The intervention effect on HM zinc was also present in the subgroup of mothers who had all four consecutive samples examined in the first 3 months (*n* = 147, data not shown).

**Table 6:** Time-weighted area-under-the-curve for zinc concentrations (μg/mL/day) in human milk samples collected in the NiPPeR study between birth and 3 months of age.

**Site**	**Sample size (intervention/control)**	**Intervention**	**Control**	**aMD**	***p*-value**
Overall	100/96	2,399 (2,252, 2,546)	2,163 (2,013, 2,314)	236 (34, 437)	**0.022**
Singapore	70/66	2,754 (2,583, 2,926)	2,473 (2,297, 2,650)	281 (33, 529)	**0.027**
New Zealand	30/30	2,083 (1,835, 2,331)	1,857 (1,608, 2,105)	226 (−132, 585)	0.211

Data are the least-squares means (i.e., adjusted means) for each group or the adjusted mean differences (aMD) and respective 95% confidence intervals, adjusted for group, visit, study site, adherence, maternal pre-pregnancy body mass index, and gestational age at birth. Bold font indicates a statistically significant difference between groups (at p < 0.05).

**Figure 3:**
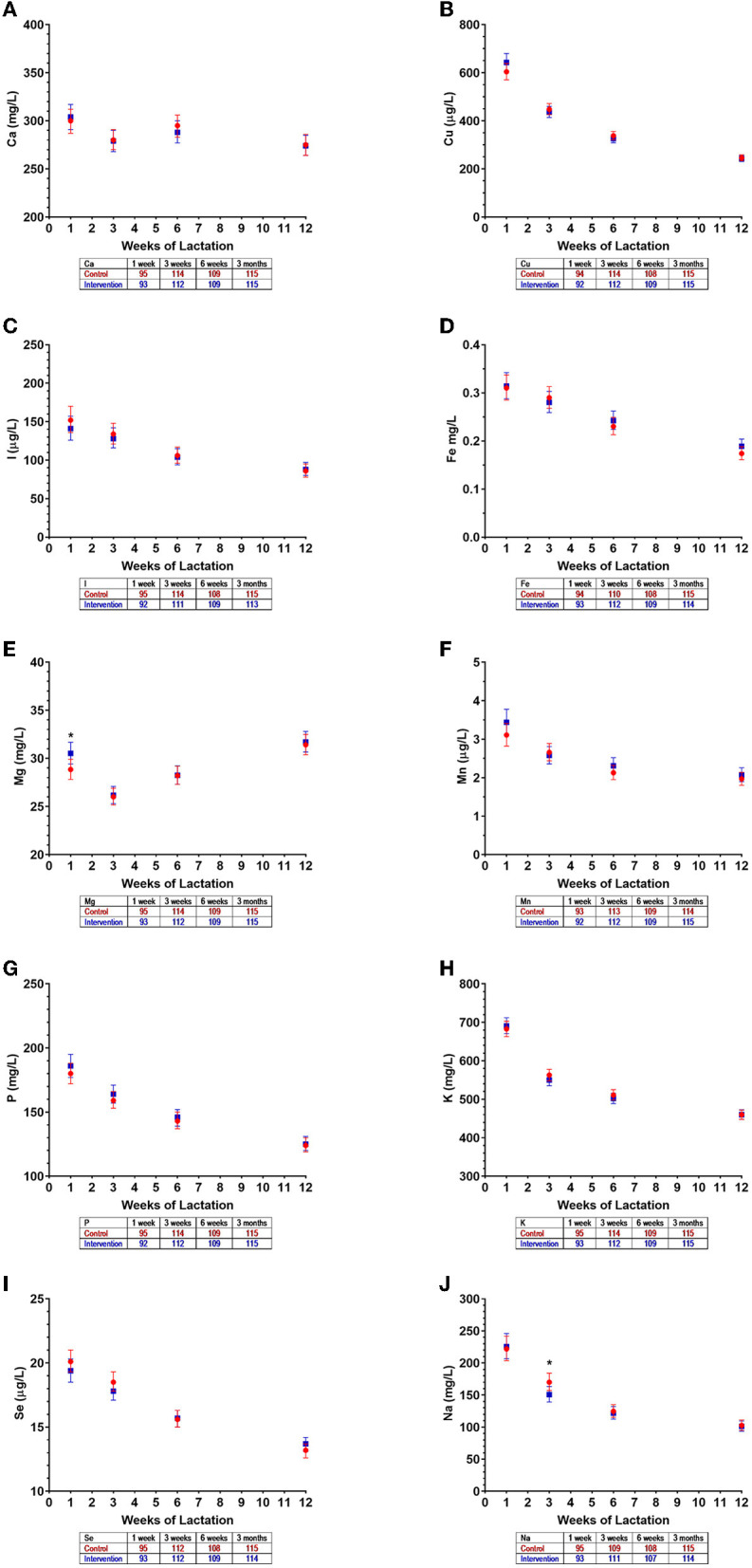
Mineral concentrations in human milk of control (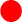) and intervention (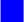) groups in the NiPPeR study during the first 3 months of lactation: **(A)** calcium, **(B)** copper, **(C)** iodine, **(D)** iron, **(E)** magnesium, **(F)** manganese, **(G)** phosphorus, **(H)** potassium, **(I)** selenium, and **(J)** sodium. Data are the least-squares means (i.e., adjusted means) for each group, adjusted for visit, an interaction term (group*visit), study site, adherence, maternal pre-pregnancy body mass index, and gestational age at birth; error bars represent the respective 95% confidence intervals. *p < 0.05 for a difference between intervention and control groups at a given time point. The number of HM samples per group analyzed at a given time point are provided in the tables below the *x* axes.

As expected, for other HM minerals not differently supplemented between the groups, there were no observed differences between the intervention and control groups in the first 3 months of lactation (Figures 3A–J). The exceptions were isolated (and likely random) findings on magnesium at 1 week (Figure 3E) and sodium at 3 weeks (Figure 3J).

In analyses stratified by site, among the 158 Singapore mothers, average zinc concentrations over the first 3 months were 15% higher in the intervention group compared to controls (*p* = 0.015), as also observed for the TwAUC (*p* = 0.027; Table 6). This difference was also most evident at 6 weeks [2,364 μg/L (95% CI 2,132, 2,621) vs. 1,919 μg/L (95% CI 1,729, 2,131), respectively; *p* = 0.006] (Figure 2B). No differences in zinc concentrations were detected in the 180 New Zealand mothers, likely due to a smaller number of participants in the first 3 months, although a similar pattern overall was observed (Figure 2C).

### 3.3. Changes in minerals over time in New Zealand (0–12 months)

**Figure 4:**
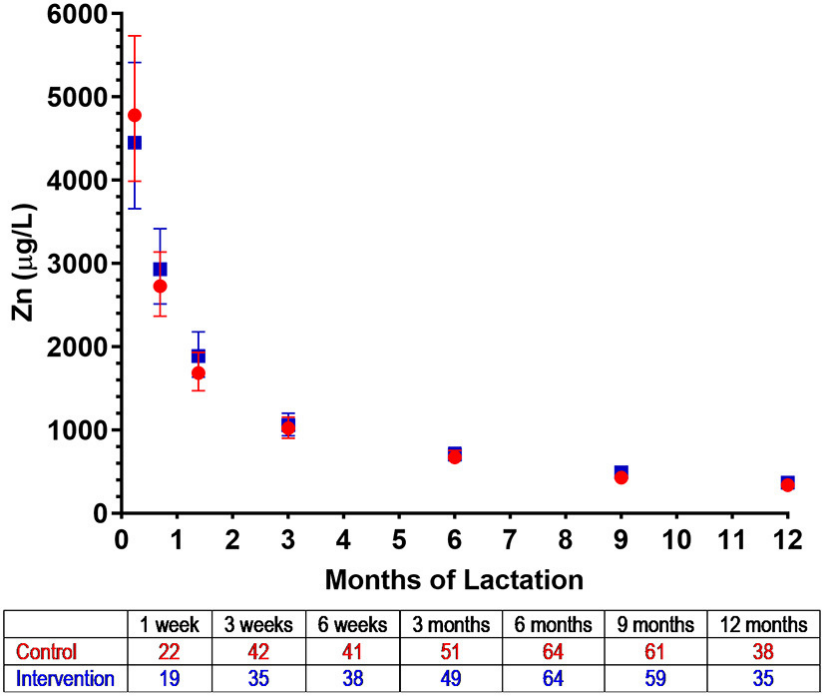
Zinc concentrations in human milk from control (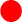) and intervention (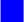) groups in New Zealand in the NiPPeR study during 12 months of lactation. Data are the least-squares means (i.e., adjusted means) for each group adjusted for visit, a group*visit interaction term, adherence, maternal pre-pregnancy body mass index, and gestational age at birth; error bars represent the respective 95% confidence intervals. The number of HM samples per group analyzed at a given time point are provided in the tables below the *x* axes.

Zinc concentrations in HM in New Zealand decreased markedly over the first 3 months of lactation and continued to decline until 12 months (Figure 4). In both control and intervention groups, zinc concentration peaked at 1 week [4,452 μg/L (95% CI 3,659, 5,416) and 4,781 μg/L (95% CI 3,987, 5,734), respectively], with an ~4.5-fold reduction by 3 months in each group [1,057 μg/L (95% CI 930, 1,201) and 1,020 μg/L (95% CI 901, 1,156), respectively] (Figure 4). Zinc concentrations continued to decline and reached a nadir at 12 months at the end of our HM collection period [340 μg/L (95% CI 392, 295) and 365 μg/L (95% CI 423, 315), respectively] (Figure 4).

**Figure 5:**
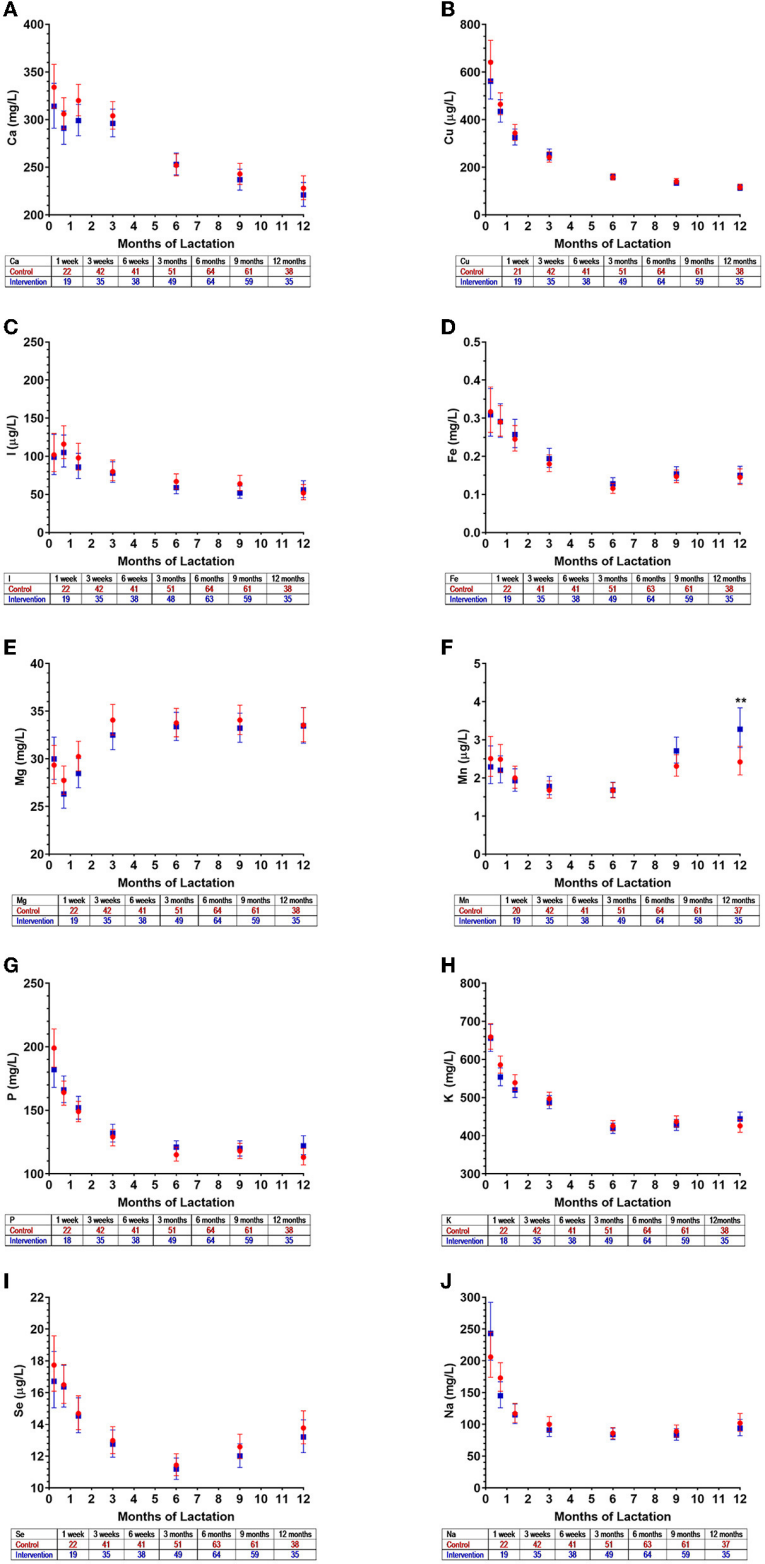
Mineral concentrations in human milk from control (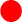) and intervention (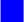) groups in New Zealand in the NiPPeR study during 12 months of lactation: **(A)** calcium, **(B)** copper, **(C)** iodine, **(D)** iron, **(E)** magnesium, **(F)** manganese, **(G)** phosphorus, **(H)** potassium, **(I)** selenium, and **(J)** sodium. Data are the least-squares means (i.e., adjusted means) for each group adjusted for visit, a group*visit interaction term, adherence, maternal pre-pregnancy body mass index, and gestational age at birth; error bars represent the respective 95% confidence intervals. **p < 0.01 for the difference between intervention and control at a given time point. The number of HM samples per group analyzed at a given time point are provided in the tables below the *x* axes.

For other minerals, different patterns of change were observed over time (Figure 5; Supplementary Table 6). Calcium and copper concentrations progressively decreased throughout 12 months of lactation, while iodine remained stable throughout the study period (Figure 5). Concentrations of iron, potassium, sodium, and phosphate gradually declined until 6 months, but then remained relatively constant until 12 months (Figure 5). In contrast, magnesium concentrations increased during the first 3 months of lactation but were largely unchanged thereafter (Figure 5). The concentrations of manganese and selenium fell over the initial months of lactation, with a nadir observed at 6 months, and increasing concentrations thereafter (Figure 5). The patterns of temporal changes in HM minerals were unchanged across all groups when assessed in a subset of New Zealand participants from whom HM samples were collected in at least five of the six visits between 3 weeks and 12 months (data not shown).

## 4. Discussion

The present study showed that the NiPPeR intervention supplement containing zinc starting before conception and taken throughout pregnancy increased subsequent HM zinc concentrations compared to a control supplement without zinc. Overall, the effect was most evident at 6 weeks and persisted throughout the first 3 months of lactation. This effect was more evident in Singapore than in New Zealand, perhaps due to a smaller sample size early in lactation at the latter site.

To the best of our knowledge, this is the first study examining the impact of zinc supplementation prior to as well as during pregnancy on HM zinc concentrations, with most previous intervention studies beginning well into the first trimester of pregnancy. A study in Indonesian women observed no effects of zinc supplementation (30 mg/day) during pregnancy (from before 20 weeks gestation until delivery) on HM zinc concentrations at the first and sixth months of lactation, although the direction of change was similar to that observed in the present study (32). In contrast, in the current study, we demonstrated a 19% increase in HM zinc concentrations at 6 weeks of lactation as a result of zinc supplementation (10 mg/day) taken before and during pregnancy. Such effects of supplementation may not have been detected in the previous study due to lack of intervention during preconception, a smaller sample size as compared to the present study, and lack of a HM collection time point at 6 weeks where we observed the peak difference. The authors of the Indonesian study acknowledged that wide variation in the single HM sample collected at any time across the first month of lactation could have masked the intervention effect (32), as zinc concentrations dynamically change during this time as we have observed (Figure 2). Nevertheless, both studies indicate that pregnancy zinc supplementation does not influence HM concentrations at 6 months of lactation, as the pregnancy intervention effect would be expected to decline postnatally over time.

Previous studies reported no associations between maternal zinc intake or plasma zinc at the time of lactation and HM zinc concentrations (2, 33–36). It has been suggested that HM zinc concentrations are maintained through mobilization of maternal zinc pools during lactation (3). For example, previous studies have observed increased intestinal zinc absorption during lactation compared to preconception (37) or early pregnancy (38). Also, active zinc transport has been suggested to be tightly regulated in the mammary gland (3, 39). Zinc transporters (ZnTs) are localized throughout the body including the intestines, bone, and the mammary gland (40). ZnT2 and ZnT4 are predominantly found in the mammary glands. Studies have identified increased gene expression of ZnT4 in mammary glands of rodents during lactation (41–43) and ZnT2 in human breast cell lines (39, 44). In the NiPPeR intervention group, the estimated cumulative exposure of zinc was 3,416 mg, considering the prescribed dose (10 mg/day), average duration, and average adherence rate (86.9%). Zinc acquired and stored in the body during preconception and pregnancy through supplementation could have contributed to HM zinc concentrations in the intervention group. For example, ~30% of total body zinc is found in bone (11) and over the first 6 months of full lactation, 4–6% of bone mass is lost, enabling maternal bone to contribute ~20% of HM zinc (45). Hence, it can be speculated that in the NiPPeR study, zinc supplementation during preconception and pregnancy led to greater storage in bone and other maternal tissues that contributed to increased HM zinc concentrations.

In addition, we have observed higher HM zinc concentrations in the Singapore site compared to the New Zealand site. We speculate this could be due to differences in dietary patterns during pregnancy, potentially resulting in different amounts of dietary zinc stored in the body that can contribute to HM zinc during lactation.

It is unknown if other factors such as smoking during pregnancy influence HM composition. In the current study, passive smoking rates during pregnancy were higher in the control group than in the intervention group. When smoking was adjusted for in our model assessing intervention effects on HM mineral concentrations (including zinc), smoking (predominantly passive) during pregnancy was not associated with HM mineral concentrations and did not alter the overall intervention effect on HM zinc (data not shown). However, mother's smoking behavior might change after delivery and unavailability of smoking rates during lactation in the current study limits our understanding of potential effects of smoking on HM minerals. Nonetheless, previous studies reported no difference in HM zinc concentrations between smoking and non-smoking mothers (46, 47). Therefore, this is expected to have limited impact on the observed intervention effect on increased HM zinc concentrations in the current study.

HM zinc concentrations progressively decline throughout lactation, which may be due to changes in transport activity or maternal dietary intake, although the latter is less likely. Previously, Silvestre et al. (48) showed that zinc concentration in unsupplemented women was the highest in colostrum at 7,990 ± 3,230 μg/L, which decreased to 1,050 ± 710 μg/L by day 90. Similarly, Djurovic et al. (45) reported a decrease in zinc concentration from 4,700 ± 1,740 μg/L at day 1 to 460 ± 360 μg/L at 6 months. These concentrations are comparable to those in our study at the respective time points. Here, beyond 6 months, HM zinc concentrations continued to steadily decrease until 12 months of lactation, which has also been described previously (49).

While previous studies have examined HM mineral concentration only for a short period of time, ranging from few weeks to 6 months of lactation, we were able to describe patterns in HM zinc and other mineral concentrations for a longer lactation period, until 12 months. Changes in HM mineral concentrations over lactation may reflect different roles of these minerals at different stages of infancy.

It was reported previously that HM calcium concentrations increase in the first 6 weeks of lactation (50, 51). We observed that not only calcium but also magnesium increases within the first 3 months then steadily decreases until 12 months of lactation. Such higher concentrations of HM calcium and magnesium may promote bone formation in early infancy (50).

As reported previously, we also observed decreases in HM copper (49, 52, 53), iron (54, 55), phosphorus (50), potassium and sodium (55–57) over the first 6 months of lactation. Beyond this time, copper concentrations steadily decreased while iron, phosphorus, potassium, and sodium concentrations remained relatively stable until 12 months of lactation. On the other hand, iodine concentrations were reported to remain constant in the first 8 weeks of lactation (58); similarly, we observed that beyond this time point, it continued to remain unchanged throughout 12 months of lactation.

There are limited studies that have investigated changes in HM manganese and selenium concentrations over time. Both were reported to decrease in the first 4 months for manganese (49) and in the first month for selenium (52). We demonstrated similar initial falls in manganese and selenium over the initial months of lactation, with a nadir at 6 months, and increasing concentrations thereafter. How these changes in HM manganese and selenium relate to the developmental stage of the infant and their implications for infant outcomes requires further investigation.

## 5. Strengths and limitations

This study investigated the impact of nutritional supplementation during preconception and pregnancy on HM mineral composition during lactation. An international, multicenter design allowed the investigation of HM mineral composition in a large population of diverse ethnic groups. The Singapore and New Zealand study sites used standardized sample collection, processing, storage and mineral quantification methods, minimizing any potential variations that might have occurred during these processes. In addition, as we have tightly controlled the visit windows, so each HM collection time point was at a distinctive stage of lactation, making it possible to describe the changes in HM mineral concentration between the different stages of lactation. However, due to logistical constraints, longitudinal samples could not be collected from every participant. To address the imbalance in the number of samples at each time point, a repeated measures design was used for statistical analyses. While maternal diet and use of other supplements during lactation was not considered in this study, previous studies reported that HM zinc concentrations are not associated with dietary zinc intake (33). Therefore, other dietary sources of zinc are expected to have limited impact on HM concentrations in the current study.

## 6. Conclusions

This study showed that maternal supplementation of zinc from as early as preconception and pregnancy influences HM zinc concentrations during lactation. This ensures not only adequate maternal zinc status during these times but also adequate zinc transfer to the infants *via* HM. In the future, ongoing evaluation of offspring from this cohort will help to understand the associations between HM zinc concentrations and both short- and long-term offspring outcomes.

## Data availability statement

The datasets presented in this article are not readily available because public sharing of the data was not part of the original participant informed consent. Requests to access the datasets should be directed to WC, w.cutfield@auckland.ac.nz.

## Ethics statement

The studies involving human participants were reviewed and approved by the study was conducted in Southampton (UK), Singapore, and Auckland (New Zealand), with ethics approval obtained at each site [Southampton—Health Research Authority National Research Ethics Service Committee South Central Research Ethics Committee (15/SC/0142); Singapore—the National Healthcare Group Domain Specific Review Board (2015/00205); and New Zealand—Northern A Health and Disability Ethics Committee (15/NTA/21)]. All participants provided written informed consent. The patients/participants provided their written informed consent to participate in this study.

## NiPPeR Study Group

The NiPPeR Study Group authors for the Frontiers in Nutrition citation comprises:

- Benjamin B Albert (b.albert@auckland.ac.nz),
- Shelia J Barton (S.J.Barton@soton.ac.uk),
- Mary Cavanagh (m.cavanagh@auckland.ac.nz),
- Hsin Fang Chang (hsin_Fang_Chang@nuhs.edu.sg),
- Yap Seng Chong (yap_seng_chong@nuhs.edu.sg),
- Mary F Chong (mary_chong@nus.edu.sg),
- Cathryn Conlon (C.Conlon@massey.ac.nz),
- Cyrus Cooper (cc@mrc.soton.ac.uk),
- Paula Costello (pc@mrc.soton.ac.uk),
- Vanessa Cox (vac@mrc.soton.ac.uk),
- Christine Creagh (christine.creagh@auckland.ac.nz),
- Marysia Depczynski (m.depczynski@auckland.ac.nz),
- Sarah El-Heis (se@mrc.soton.ac.uk),
- Judith Hammond (j.hammond@auckland.ac.nz),
- Nicholas C Harvey (nch@mrc.soton.ac.uk),
- Mrunalini Jagtap (mrunalini_jagtap@sics.a-star.edu.sg),
- Timothy Kenealy (t.kenealy@auckland.ac.nz),
- Heidi Nield (hn@mrc.soton.ac.uk),
- Justin M O'Sullivan (justin.osullivan@auckland.ac.nz),
- Gernalia Satianegara (gernalia_satianegara@sics.a-star.edu.sg),
- Irma Silva-Zolezzi (Irma.SilvaZolezzi@rdls.nestle.com),
- Shu E Soh (shu_e_soh@nuhs.edu.sg),
- Vicky Tay (Vicky_tay@sics.a-star.edu.sg),
- Rachael Taylor (rachael.taylor@otago.ac.nz),
- Elizabeth Tham (elizabeth_tham@nuhs.edu.sg),
- Philip Titcombe (pt6g13@soton.ac.uk),
- Clare Wall (c.wall@auckland.ac.nz),
- Ray Wong (ray_wong@sics.a-star.edu.sg),
- Gladys Woon (gladys_woon@nuhs.edu.sg).

## Author contributions

KMG, S-YC, and WSC led the design of the original study. The present sub-study was developed and undertaken by SMH, SDe, JGBD, MHV, FH, SKT, and WSC. SDu supervised the laboratory analyses. SMH and JGBD performed the statistical analyses. SMH led the manuscript writing. SDe contributed to sections of the manuscript. SKT and WSC supervised all aspects of the present study. All authors contributed to interpretation, manuscript revision, read, and approved the final version.
