# Supplementary material for: A nutritional supplement containing zinc during preconception and pregnancy increases human milk zinc concentrations
Source: Front Nutr. 2023 Jan 10;9:1034828. doi: 10.3389/fnut.2022.1034828 (PMC9872665; doi:10.3389/fnut.2022.1034828)
Supplement: Supplementary file 1 [file Table_1.docx]

Supplementary Material

**Supplementary Table 1.** The inclusion, exclusion, and withdrawal criteria for participants in the NiPPeR study (adapted from Godfrey et al. Trials 2017;18:131).

| **Inclusion criteria** | **Exclusion criteria** | **Withdrawal criteria** |
| --- | --- | --- |
| - Women aged 18-38 years - Living in Southampton, Singapore, or Auckland - In Southampton and Auckland, planning to have future maternity care in Southampton and Auckland, respectively - In Singapore, willing to deliver at the National University Hospital - Planning to conceive within 6 months (although conception up to 12 months after phenotyping deemed acceptable) - In Singapore, being of Chinese, Malay, or Indian ethnicity, or of mixed Chinese/Malay/Indian ethnicity - Ability to provide written informed consent | - Pregnant or currently breastfeeding - Assisted fertility apart from those taking clomiphene or letrozole alone - Pre-existing type 1 or type 2 diabetes (defined as a fasting plasma glucose concentration ≥7.0 mmol/L or post OGTT 2-h plasma glucose concentration ≥11.1 mmol/L) - Oral or implanted contraception currently or in the last month, or with an intrauterine contraceptive device in situ - Use of metformin or systemic steroids currently or in the last month - Use of anticonvulsant medication currently or in the last month - Treatment for HIV, Hepatitis B or C, currently or in the last month - Known serious food allergy | - Participant wishes to withdraw from the study - Participant unwilling or unable to comply with the study protocol (including attendance at study visits, undergoing clinical assessments, or biosampling) - An overall uptake level of intervention/control nutritional drink of less than 60% evidenced by sachet counting, each case reviewed by the investigators. - Pregnancy before or at preconception visit 2 - Miscarriage (pregnancy loss before 24 weeks’ gestation) or ectopic pregnancy. In the event of a first-trimester pregnancy loss, participant could re-join the study with the same randomisation code - Multiple pregnancy (twins or other multiples) - Infant death in the perinatal period (for post-birth secondary outcomes) - Any adverse reaction deemed by the investigators to be causally related to the intervention - Withdrawal at the discretion of the investigators for medical reasons |

**Supplementary Table 2.** Number of participants according to reason for exclusion from the NiPPeR study by site.

| **Study phase** | **Reason for exclusion** | **Control** | | **Intervention** | |
| --- | --- | --- | --- | --- | --- |
|  |  | **Singapore** | **New Zealand** | **Singapore** | **New Zealand** |
| **Pregnancy** | Miscarriage (≤ 24 weeks gestation) | 20 | 14 | 18 | 21 |
|  | Twin pregnancy | 1 | 1 | 1 | 2 |
|  | Ectopic pregnancy | 1 | 2 | 1 |  |
|  | Termination^*^ | 2 | 1 |  |  |
|  | Other medical reasons |  | 1 |  | 1 |
|  | Lost to follow-up | 2 | 2 | 3 | 2 |
| **Birth** | Lost to follow-up | 2 | 1 | 2 |  |
|  | Infant’s medical reasons |  | 1 |  | 1 |
|  | Stillbirth |  |  |  | 1 |

^*^ Includes one case of Klinefelter syndrome and other unknown reasons.

**Supplementary Table 3.** Number of values for each mineral measured in human milk in the NiPPeR study, whose concentrations were below the lower limit of quantification (LLoQ) or classified as extreme values.

| **Mineral** | **< LLoQ** | **< Mean – 5 SD** | **> Mean + 5 SD** |
| --- | --- | --- | --- |
| Zinc | nil | nil | nil |
| Calcium | nil | nil | nil |
| Cobalt | 490 (41.4%) | n/a | n/a |
| Copper | nil | nil | 3 (0.25 %) |
| Iodine | nil | nil | 6 (0.50%) |
| Iron | nil | nil | 8 (0.68 %) |
| Magnesium | nil | nil | nil |
| Manganese | 2 (0.17%) | nil | 7 (0.59%) |
| Nickel | 983 (83.0%) | n/a | n/a |
| Phosphorus | nil | nil | 1 (0.08%) |
| Potassium | nil | nil | nil |
| Selenium | nil | nil | 5 (0.42%) |
| Sodium | nil | nil | 12 (1.01%) |

n/a, not applicable; SD, standard deviation.

**Supplementary Table 4.** Baseline and perinatal characteristics of participants in the NiPPeR study who provided at least one human milk sample during 12 months of lactation by site.

|  | **Singapore (n = 158)** | | **New Zealand (n = 180)** | |
| --- | --- | --- | --- | --- |
|  | **Control** | **Intervention** | **Control** | **Intervention** |
| n | 78 (49.4%) | 80 (50.6%) | 92 (51.1%) | 88 (48.9%) |
| Adherence (%) | 87.2 ± 12.4 | 85.5 ± 14.9 | 87.7 ± 10.2 | 88.2 ± 11.9 |
| Duration of supplementation (days) | 390.9 ± 102.8 | 398.9 ± 100.1 | 416.3 ± 105.9 | 387.8 ± 96.3 |
| Age at delivery(years) | 32.7 ± 3.3 | 31.6 ± 2.8 | 32.2 ± 3.0 | 32.7 ± 3.3 |
| Maternal pre-pregnancy BMI (kg/m^2^) | 24.5 ± 4.9 | 23.5 ± 5.3 | 25.1 ± 5.0 | 24.5 ± 4.9 |
| Ethnicity |  |  |  |  |
| Caucasian | – | – | 70 (76.1%) | 67 (76.1%) |
| Chinese | 62 (79.5%) | 62 (77.5%) | 8 (8.7%) | 7 (8.0%) |
| South Asian | 5 (6.4%) | 7 (8.8%) | 5 (5.4%) | 3 (3.4%) |
| Malay | 10 (12.8%) | 10 (12.5%) | – | – |
| Other | 1 (1.3%) | 1 (1.3%) | 9 (9.8%) | 11 (12.5%) |
| Maternal pre-pregnancy BMI status |  |  |  |  |
| Underweight or Normal weight | 45 (57.7%) | 46 (57.5%) | 55 (59.8%) | 57 (64.8%) |
| Overweight | 18 (23.1%) | 29 (36.3%) | 23 (25.0%) | 19 (21.6%) |
| Obesity | 15 (19.2%) | 4 (5.0%) | 14 (15.2%) | 12 (13.6%) |
| Missing | – | 1 (1.3%) | – | – |
| Highest level of education | | | | |
| Bachelor’s degree or higher | 61 (78.2%) | 64 (80.0%) | 76 (82.6%) | 72 (81.8%) |
| Lesser qualification* | 17 (21.8%) | 16 (20.0%) | 16 (17.4%) | 16 (18.2%) |
| Household income quintile | | | | |
| 5 (lowest) | 3 (3.8%) | 1 (1.3%) | 1 (1.1%) | – |
| 4 | 10 (12.8%) | 13 (16.3%) | 2 (2.2%) | 3 (3.4%) |
| 3 | 25 (32.1%) | 28 (35.0%) | 19 (20.7%) | 15 (17.0%) |
| 2 | 24 (30.8%) | 24 (30.0%) | 36 (39.1%) | 31 (35.2%) |
| 1 (highest) | 13 (16.7%) | 11 (13.8%) | 31 (33.7%) | 32 (36.4%) |
| Missing | 3 (3.8%) | 3 (3.8%) | 3 (3.3%) | 7 (8.0%) |
| Smoking during pregnancy | | | | |
| None | 58 (74.4%) | 66 (82.5%) | 76 (82.6%) | 82 (94.3%) |
| Passive | 19 (24.4%) | 11 (13.8%) | 14 (15.2%) | 5 (5.7%) |
| Active | 1 (1.3%) | 3 (3.8%) | 2 (2.2%) | – |
| Missing | – | – | – | 1 (1.3%) |
| GDM | | | | |
| No GDM | 52 (66.7%) | 51 (63.7%) | 74 (80.4%) | 74 (84.1%) |
| GDM | 26 (33.3%) | 29 (36.3%) | 16 (17.4%) | 14 (15.9%) |
| Missing | – | – | 2 (2.2%) | – |
| Hypertension/pre-eclampsia |  |  |  |  |
| No | 77 (98.7%) | 79 (98.8%) | 90 (97.8%) | 86 (97.7%) |
| Yes | – | 1 (1.3%) | 2 (2.2%) | 2 (2.3%) |
| Missing | 1 (1.3%) | – | – | – |
| Mode of Delivery | | | | |
| Vaginal delivery | 63 (80.8%) | 60 (75.0%) | 62 (67.4%) | 59 (67.0%) |
| Caesarean section | 14 (17.9%) | 20 (25.0%) | 30 (32.6%) | 29 (33.0%) |
| Missing | 1 (1.3%) | – | – | – |
| Infant gestational age |  |  |  |  |
| Gestational age (weeks) | 39.7 ± 1.4 | 38.8 ± 1.5 | 39.5 ± 1.6 | 39.7 ± 1.4 |
| Preterm | 6 (7.7%) | 8 (10.0%) | 8 (8.7%) | 3 (3.4%) |
| Term or Post-term | 72 (92.3%) | 72 (90.0%) | 84 (91.3%) | 85 (96.6%) |
| Infant birth weight |  |  |  |  |
| Birth weight (kg) | 3.02 ± 0.45 | 2.97 ± 0.42 | 3.42 ± 0.54 | 3.47 ± 0.50 |
| Appropriate for gestational age | 66 (84.6%) | 66 (82.5%) | 78 (84.8%) | 77 (87.5%) |
| Small for gestational age | 11 (14.1%) | 14(17.5%) | 5 (5.4%) | 5 (5.7%) |
| Large for gestational age | 1 (1.3%) | – | 9 (9.8%) | 6 (6.8%) |
| Parity | | | | |
| Primiparous | 48 (61.5%) | 35 (43.8%) | 66 (71.7%) | 60 (68.2%) |
| Multiparous | 30 (38.5%) | 45 (56.3%) | 26 (28.3%) | 28 (31.8%) |
| Infant sex | | | | |
| Male | 41 (52.6%) | 37 (46.3%) | 35 (38.0%) | 42 (47.7%) |
| Female | 37 (47.4%) | 43 (53.8%) | 57 (62.0%) | 46 (52.3%) |

Data are n (%) or mean ± standard deviation (SD). Adherence to the study protocol was determined by sachet counting. Duration of supplementation calculated by number of days from randomisation date to delivery date. Body mass index (BMI) status was defined using ethnic-specific thresholds for BMI categories: for Asians, under or normal weight <23.0 kg/m^2^, overweight 23.0–27.49 kg/m^2^, obesity ≥27.5 kg/m^2^; for non-Asians, under or normal weight <25.0 kg/m^2^, overweight 25.0–29.99 kg/m^2^, obesity ≥30.0 kg/m^2^. Gestational diabetes (GDM) was defined by International Association of Diabetes and Pregnancy Study Groups criteria (Diabetes Care 2010;33:676-82). Gestational age was determined using a pre-specified algorithm as previously described (Thorax 2010;65:1099–106) with preterm defined as birth <37 weeks of gestation, and term or post-term as birth at ≥37 weeks of gestation. Breastfeeding duration in weeks determined by age last fed direct or expressed human milk. * Including incomplete and complete high school qualifications, and other tertiary level qualifications below bachelors (e.g., diploma or certificate).

**Supplementary Table 5.** Baseline and perinatal characteristics of Singapore and New Zealand participants in the NiPPeR study who continued to postpartum stage.

|  | **Overall (n = 387)** | |
| --- | --- | --- |
|  | **Control** | **Intervention** |
| n | 194 (50.1%) | 193 (49.9%) |
| Adherence (%) | 87.7 11.4 | 86.8 13.2 |
| Duration of supplementation (days) | 401 ± 105 | 389 ± 97 |
| Age at delivery (years) | 32.0 3.0 | 32.4 3.2 |
| Maternal pre-pregnancy BMI (kg/m^2^) | 24.6 5.4 | 23.7 4.7 |
| Ethnicity |  | |
| Caucasian | 80 (41.2%) | 85 (44.0%) |
| Chinese | 71 (36.6%) | 70 (36.3%) |
| South Asian | 11 (5.7%) | 14 (7.3%) |
| Malay | 11 (5.7%) | 11 (5.7%) |
| Other | 21 (10.8%) | 13 (6.7%) |
| Maternal pre-pregnancy BMI status |  | |
| Underweight or normal weight | 113 (58.2%) | 116 (60.1%) |
| Overweight | 45 (23.2%) | 56 (29.0%) |
| Obesity | 36 (18.6%) | 20 (10.4%) |
| Missing | – | 1 (0.5%) |
| Highest level of education |  | |
| Bachelor’s degree or higher | 154 (79.4%) | 152 (78.8%) |
| Lesser qualification* | 40 (20.6%) | 41 (21.2%) |
| Household income quintile |  | |
| 5 (lowest) | 4 (2.1%) | 1 (0.5%) |
| 4 | 14 (7.2%) | 16 (8.3%) |
| 3 | 53 (27.3%) | 45 (23.3%) |
| 2 | 70 (36.1%) | 65 (33.7%) |
| 1 (highest) | 47 (24.2%) | 53 (27.5%) |
| Missing | 6 (3.1%) | 13 (6.7%) |
| Smoking during pregnancy |  | |
| None | 151 (77.8%) | 171 (88.6%) |
| Passive | 38 (19.6%) | 17 (8.8%) |
| Active | 4 (2.1%) | 4 (2.1%) |
| Missing | 1 (0.5%) | 1 (0.5%) |
| GDM |  | |
| No GDM | 141 (73.4%) | 141 (73.1%) |
| GDM | 51 (26.6%) | 52 (26.9%) |
| Missing | 2 (1.0%) | – |
| Hypertension/pre-eclampsia |  |  |
| No | 190 (97.9%) | 190 (98.4%) |
| Yes | 3 (1.5%) | 3 (1.6%) |
| Missing | 1 (0.5%) | – |
| Mode of delivery |  | |
| Vaginal delivery | 139 (71.6%) | 138 (71.5%) |
| Caesarean section | 54 (27.8%) | 55 (28.5%) |
| Missing | 1 (0.5%) | – |
| Infant gestational age |  |  |
| Gestational age (weeks) | 39.2 1.7 | 39.3 1.5 |
| Preterm | 17 (8.8%) | 12 (6.2%) |
| Term or post-term | 177 (91.2%) | 181 (93.8%) |
| Infant birth weight |  |  |
| Birth weight (kg) | 3.25 ± 0.56 | 3.28 ± 0.53 |
| Appropriate for gestational age | 164 (85.5%) | 164 (85.0%) |
| Small for gestational age | 18 (9.3%) | 19 (9.8%) |
| Large for gestational age | 12 (6.2%) | 10 (5.2%) |
| Parity |  | |
| Primiparous | 134 (69.1%) | 109 (56.5%) |
| Multiparous | 60 (30.9%) | 84 (43.5%) |
| Infant sex |  | |
| Male | 92 (47.4%) | 91 (47.2%) |
| Female | 102 (52.6%) | 102 (52.8%) |

Data are n (%) or mean ± standard deviation (SD). Adherence to the study protocol was determined by sachet counting. Duration of supplementation calculated by number of days from randomisation date to delivery date. Body mass index (BMI) status was defined using ethnic-specific thresholds for BMI categories: for Asians, under or normal weight <23.0 kg/m^2^, overweight 23.0–27.49 kg/m^2^, obesity ≥27.5 kg/m^2^; for non-Asians, under or normal weight <25.0 kg/m^2^, overweight 25.0–29.99 kg/m^2^, obesity ≥30.0 kg/m^2^. Gestational diabetes (GDM) was defined by International Association of Diabetes and Pregnancy Study Groups criteria (Diabetes Care 2010;33:676-82). Gestational age was determined using a pre-specified algorithm as previously described (Thorax 2010;65:1099–106) with preterm defined as birth <37 weeks of gestation, and term or post-term as birth at ≥37 weeks of gestation. Breastfeeding duration in weeks determined by age last fed direct or expressed human milk. * Including incomplete and complete high school qualifications, and other tertiary level qualifications below bachelors (e.g., diploma or certificate).

**Supplementary Table 6.** Overall mineral concentrations in human milk of participants in New Zealand in the NiPPeR study, during 12 months of lactation.

| **Mineral** | **1 week** | **3 weeks** | **6 weeks** | **3 months** | **6 months** | **9 months** | **12 months** |
| --- | --- | --- | --- | --- | --- | --- | --- |
| Zinc (μg/L) | 4653 ± 1327 | 2935 ± 1018 | 1939 ± 839 | 1165 ± 545 | 790 ± 401 | 563 ± 360 | 452 ± 344 |
| Calcium (mg/L) | 349 ± 77 | 312 ± 82 | 329 ± 79 | 311 ± 66 | 260 ± 38 | 243 ± 34 | 231 ± 36 |
| Copper (μg/L) | 616 ± 164 | 455 ± 101 | 340 ± 80 | 251 ± 63 | 176 ± 71 | 152 ± 63 | 126 ± 46 |
| Iodine (μg/L) | 110 ± 61 | 131 ± 87 | 110 ± 77 | 91 ± 55 | 78 ± 66 | 74 ± 58 | 71 ± 53 |
| Iron (mg/L) | 0.33 ± 0.13 | 0.32 ± 0.14 | 0.28 ± 0.16 | 0.2 ± 0.07 | 0.14 ± 0.08 | 0.16 ± 0.08 | 0.16 ± 0.08 |
| Magnesium (mg/L) | 30.6 ± 5.4 | 27.9 ± 6.1 | 30.1 ± 5.3 | 34.1 ± 5.1 | 34.1 ± 5.9 | 33.9 ± 7 | 33.9 ± 8.2 |
| Manganese (μg/L) | 2.68 ± 1.65 | 2.73 ± 1.46 | 2.11 ± 1.03 | 1.84 ± 0.83 | 1.9 ± 1.09 | 2.93 ± 2.24 | 3.19 ± 2.28 |
| Phosphorus (mg/L) | 195 ± 43 | 169 ± 38 | 155 ± 31 | 133 ± 24 | 120 ± 20 | 122 ± 22 | 121 ± 23 |
| Potassium (mg/L) | 655 ± 70 | 581 ± 85 | 536 ± 65 | 497 ± 55 | 426 ± 55 | 436 ± 62 | 437 ± 75 |
| Selenium (μg/L) | 17.7 ± 3.1 | 16.6 ± 3.0 | 15.1 ± 3.0 | 13.1 ± 2.9 | 11.5 ± 2.7 | 12.9 ± 4.5 | 14.1 ± 5.0 |
| Sodium (mg/L) | 253 ± 119 | 182 ± 124 | 130 ± 70 | 101 ± 39 | 90 ± 39 | 104 ± 103 | 108 ± 62 |

Data are means ± standard deviations.
